# Associations between NIHSS sub-item scores and prognosis and intracranial hemorrhage after endovascular therapy for acute anterior circulation ischemic stroke

**DOI:** 10.3389/fneur.2023.1320055

**Published:** 2024-01-08

**Authors:** Shuang Qi, Mingchao Shi, Chao Li, Kangjia Song, Jie Zhou, Feixue Yue, Wenbin Zhang, Shouchun Wang

**Affiliations:** Department of Neurology and Neuroscience, The First Hospital of Jilin University, Changchun, Jilin, China

**Keywords:** NIHSS, endovascular therapy, prognosis, intracranial hemorrhage, acute anterior circulation ischemic stroke

## Abstract

**Purpose:**

The relationship between sub-item scores on the National Institutes of Health Stroke Scale (NIHSS) scores and prognosis and intracranial hemorrhage in patients with acute ischemic stroke undergoing endovascular treatment (EVT) has been insufficiently studied. The aim of this study was to investigate the correlation between NIHSS sub-item scores, prognosis, and intracranial hemorrhage.

**Methods:**

This study included patients with acute anterior circulation ischemic stroke undergoing EVT between February 2019 and April 2022. The outcomes included functional independence, defined as a modified Rankin Scale (mRS) score ≤ 2 at 3 months after EVT, intracranial hemorrhage within 24 h after EVT, and mortality within 3 months. A multivariate regression analysis was performed, including NIHSS sub-item scores and other adjusted variables.

**Results:**

A total of 568 patients were enrolled. Of the 568 patients, 239 (45%) achieved functional independence at 3 months after EVT. The median age in this group was 63 years (IQR 52–69) and 176 (73.6%) were male patients. Intracranial hemorrhage within 24 h after EVT occurred in 170 (30%) patients. The median age in this group was 65 years (IQR 56–71) and 105 (61.8%) were male patients. In a multivariate analysis adjusted for age, gender, and factors with a value of p of <0.05, the NIHSS limb movement sub-item score was strongly associated with prognosis at 3 months (OR 0.833, 95% CI 0.758–0.915) and intracranial hemorrhage within 24 h after EVT (OR 1.161, 95% CI 1.037–1.300).

**Conclusion:**

Higher limb movement sub-item scores on the NIHSS were independently associated with a poorer prognosis at 3 months and a higher rate of intracranial hemorrhage within 24 h after EVT among patients with acute anterior circulation ischemic stroke.

## Introduction

Endovascular treatment (EVT) has become a standard treatment for patients with acute ischemic stroke (AIS) with large vessel occlusion in the anterior cerebral circulation (1–8). Patients undergoing EVT have a better prognosis, with 46% achieving functional independence (modified Rankin Scale score ≤ 2) compared to 26.5% with standard care (6). However, over half of patients still cannot walk unaided after EVT. Furthermore, futile reperfusion can occur despite successful recanalization (9).

Previous studies have identified a high baseline National Institutes of Health Stroke Scale (NIHSS) score and intracranial hemorrhage as independent factors for poor prognosis after EVT (10). Additionally, hemorrhagic transformation is associated with an increased risk of clinical complications, longer hospitalization, mortality, and poorer clinical outcomes at discharge (11). Consequently, identifying factors associated with intracranial hemorrhage and timely adjustment of the treatment plan could reduce intracranial hemorrhage rates and improve patient outcomes after EVT.

Currently, the National Institutes of Health Stroke Scale (NIHSS) is commonly assessed in AIS patients prior to EVT. The prominent role of the NIHSS in cerebrovascular diseases is well-established. The 15-item NIHSS evaluates domains including consciousness, eye movements, visual fields, motor function, sensation, coordination, language, and neglect, providing a measure of stroke severity (12). Several studies have demonstrated an association between total NIHSS scores and prognosis after EVT in AIS (13, 14). However, few studies have examined the correlation between individual NIHSS sub-item scores and patient prognosis.

This study aimed to investigate correlations between NIHSS sub-item scores and prognosis and intracranial hemorrhage in AIS patients with anterior circulation occlusion undergoing EVT and to provide evidence to help emergency clinicians rapidly identify candidates for EVT.

## Methods

### Patient selection

This retrospective study included patients with acute anterior circulation ischemic stroke undergoing EVT from February 2019 to April 2022. The study protocol was approved by the ethics committee of the First Hospital of Jilin University, which waived the requirement for informed consent due to the anonymous analysis of clinical data. As a retrospective medical record review, this study presented minimal risk to patients and did not impact clinical decision-making or diagnoses. Deidentified data were recorded by the study investigators to maintain confidentiality. The study procedures conformed to the ethical standards of the Declaration of Helsinki. The inclusion criteria were: (1) diagnosed with acute anterior circulation ischemic stroke and underwent EVT; (2) informed consent provided by the patients or their legal representative; and (3) artery occlusion in the anterior circulation confirmed by computed tomography angiography or digital subtraction angiography. The exclusion criteria were: (1) definite diagnosis of hemorrhagic stroke; (2) severe systemic illness precluding EVT tolerance; (3) Alberta Stroke Program Early CT Score (ASPECTS) <6; (4) pre-stroke modified Rankin Scale (mRS) >2; and (5) incomplete or missing data. We included patients with a baseline NIHSS score of less than 13 (median) and analyzed the effects of low and high motor scores (< 7 or ≥ 7 on motor scores) on prognosis and intracranial hemorrhage in these patients using a sensitivity analysis.

### Treatments

Intravenous thrombolysis was given to patients within 4.5 h of onset and without contraindications to intravenous thrombolysis (15). Patients undergoing EVT underwent a procedure that included a stent retriever device, aspiration, balloon dilatation, stenting, intra-arterial thrombolysis, or a combination of the above approaches (16, 17). The modality of EVT was determined by an experienced surgeon. The mode of anesthesia was local anesthesia, intravenous sedation, or general anesthesia, according to the patient’s condition and degree of cooperation. Computed tomography of the head was performed immediately and 24 h after the procedure, and magnetic resonance imaging of the head was performed approximately 3 days after the EVT.

### Data collection and definitions

The following clinical data were collected: age, gender, vascular risk factors (hypertension, diabetes, and atrial fibrillation), time from symptom onset to arterial puncture, time from puncture to reperfusion or procedure completion, Trial of Org 10,172 in Acute Stroke Treatment (TOAST) classification, baseline NIHSS score, baseline ASPECTS, and expanded Thrombolysis in Cerebral Infarction (eTICI) scale score at procedure completion. Successful recanalization was defined as eTICI grade 2b-3.

The primary outcome was the mRS score at 3 months after EVT. Functional independence was defined as mRS ≤ 2. A secondary outcome was intracranial hemorrhage within 24 h after EVT and mortality within 3 months. Any intracranial hemorrhage was recorded, including asymptomatic or symptomatic subarachnoid hemorrhage, hemorrhagic infarction, and parenchymal hematoma (10). Symptomatic intracranial hemorrhage was defined as a new intracranial hemorrhage confirmed on computed tomography or magnetic resonance imaging with a NIHSS score of ≥4 points or ≥ 2 points in one category before worsening. Outcomes were assessed by two neurologists independently. In the case of inconsistent evaluations, a third investigator evaluated and confirmed the results.

### Statistical analysis

All statistical analyses were performed using IBM SPSS Statistics 27 (IBM Corp, Armonk, NY). The normality of continuous variables was assessed with the Kolmogorov–Smirnov test. Non-normally distributed data were reported as median and interquartile range. Categorical variables were reported as frequency and percentage. Continuous variables were compared between groups using the Mann–Whitney U-test. Categorical variables were compared with the chi-square test. Multivariate binary logistic regression examined associations between NIHSS sub-item scores and outcomes, adjusting for age, sex, and variables with a *p*-value of <0.05. Adjusted variables for prognosis models were age, sex, diabetes, time to reperfusion, eTICI grade, baseline ASPECTS, and NIHSS sub-items for consciousness, gaze, motor, and language. Adjusted variables for hemorrhage models were age, sex, diabetes, atrial fibrillation, time to reperfusion, TOAST classification, baseline ASPECTS, and NIHSS motor and gaze sub-items. Adjusted variables for mortality models were age, sex, time to reperfusion, eTICI grade, and NIHSS sub-items for consciousness, gaze, facial palsy, motor, and language. Statistical significance was defined as a *p*-value of <0.05. R software (version 4.3.0) was used to generate outcome prediction curves based on motor sub-item scores.

## Results

### Characteristics of patients

**Figure 1:**
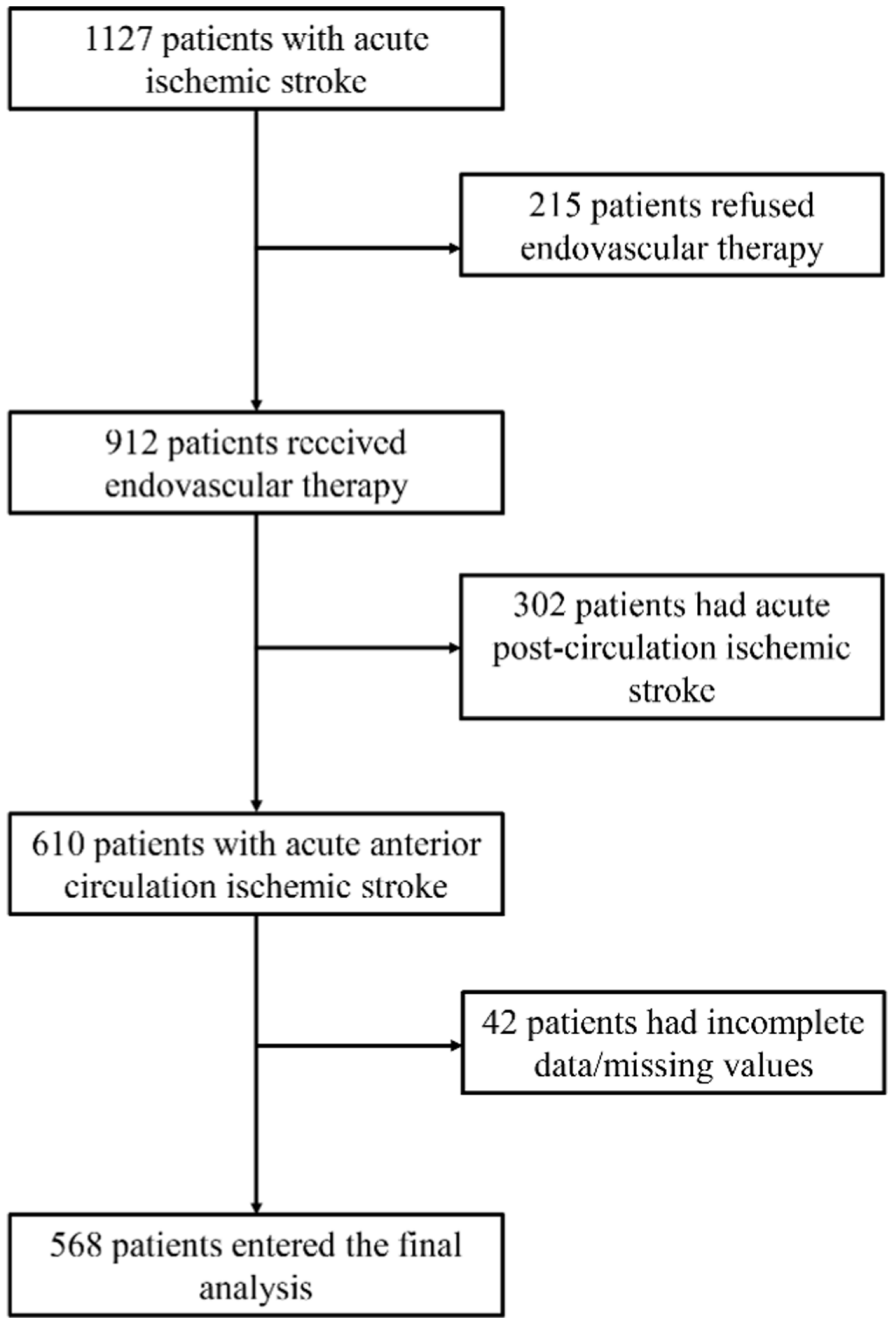
Flowchart of patient selection.

This retrospective study included 568 patients with acute anterior circulation ischemic stroke who underwent EVT at the First Hospital of Jilin University from February 2019 to April 2022. The flowchart of patient selection is shown in Figure 1. Of the 568 patients, 172 (30.3%) were female patients, the median age was 63 years [interquartile range (IQR) 54–70], and the median NIHSS score was 13 (IQR 10–16). At 3 months post-EVT, 239 patients (42.1%) achieved functional independence [modified Rankin Scale (mRS) score ≤ 2], and 329 (57.9%) did not (mRS >2) (Table 1). A total of 20 patients (3.5%) had symptomatic intracranial hemorrhage, while 150 (26.4%) had asymptomatic intracranial hemorrhage. A total of 64 patients (11.27%) died within 3 months. The baseline characteristics of the groups are compared in Table 1.

**Table 1:** Baseline demographic and clinical characteristics of patients between different outcome groups.

Characteristic	Functional independence	Non-functional independence	*p*	No intracranial hemorrhage	Intracranial hemorrhage	*p*	Mortality	Survival	*p*
*Demographic*									
Age, years	63.00 (52.00–69.00)	64.00 (55.00–70.00)	0.164	62.00 (53.00–69.00)	65.00 (56.00–71.00)	0.015	65.0 (56.00–72.20)	63.0 (54.00–69.00)	0.156
Gender [Men, n (%)]	176 (73.6)	220 (66.9)	0.083	291 (73.1)	105 (61.8)	0.007	37 (57.8)	359 (71.2)	0.040
Vascular risk factors									
Hypertension, n (%)	129 (54.0)	203 (61.7)	0.065	236 (59.3)	96 (56.5)	0.531	42 (65.6)	290 (57.5)	0.271
Diabetes, n (%)	50 (20.9)	95 (28.9)	0.032	98 (24.6)	47 (27.6)	0.449	18 (28.1)	127 (25.2)	0.724
Atrial fibrillation, n (%)	44 (18.4)	63 (19.1)	0.824	61 (15.3)	46 (27.1)	0.001	16 (25.0)	91 (18.1)	0.243
*Process measures*									
Time from onset to arterial puncture, min	360.00 (240.00–662.00)	395.00 (250.00–700.00)	0.212	373.50 (235.00–695.50)	381.50 (265.00–630.00)	0.579	337.00 (234.00–511.00)	380.00 (250.00–698.00)	0.233
Time from arterial puncture to reperfusion or procedure completion, min	70.00 (50.00–100.00)	85.00 (60.00–118.50)	<0.001	75.00 (50.00–105.00)	85.00 (65.00–120.00)	<0.001	96.00 (70.80–135.00)	75.00 (55.00–105.00)	<0.001
TOAST, n (%)			0.193			0.001*			0.582*
LAA	117 (49.0)	177 (53.8)		217 (54.5)	77 (45.3)		31 (48.4)	263 (52.2)	
CE	57 (23.8)	63 (19.1)		69 (17.3)	51 (30.0)		16 (25.0)	104 (20.6)	
SAO	4 (1.7)	8 (2.4)		12 (3.0)	0 (0.0)		0 (0.00)	12 (2.38)	
ODC	11 (4.6)	26 (7.9)		23 (5.8)	14 (8.2)		6 (9.38)	31 (6.15)	
UE	50 (20.9)	55 (16.7)		77 (19.3)	28 (16.5)		11 (17.2)	94 (18.7)	
Intravenous thrombolysis, n (%)	30 (12.6)	37 (11.2)	0.634	47 (11.8)	20 (11.8)	0.988	7 (10.9)	60 (11.9)	0.984
eTICI≥2b, n (%)	214 (89.5)	252 (76.6)	<0.001	334 (83.9)	132 (77.6)	0.074	43 (67.2)	423 (83.9)	0.002
Baseline total NIHSS score	11.00 (9.00–14.00)	13.00 (11.00–16.00)	<0.001	12.00 (9.00–15.00)	13.00 (11.00–16.00)	0.010	16.00 (13.00–19.00)	12.00 (9.75–15.00)	<0.001
Baseline ASPECTS score	10.00 (8.00–10.00)	9.00 (7.00–10.00)	<0.001	9.00 (8.00–10.00)	9.00 (7.00–10.00)	<0.001	9.00 (7.00–10.00)	9.00 (8.00–10.00)	0.053

ASPECTS, Alberta Stroke Program Early CT Score; eTICI, expanded Treatment in Cerebral Ischemia; NIHSS, National Institutes of Health Stroke Scale; sICH, symptomatic intracranial hemorrhage; TOAST, trial of org 10,172 in acute stroke treatment; LAA, large artery atherosclerosis; SAO, small artery occlusion; CE, cardioembolism; ODC, other determined cause; UE, undetermined etiology. *, Fisher’s precise test.

### Comparison of the NIHSS sub-item scores for the different groups

Based on 3-month mRS scores, the patients were categorized into functional independence and non-independence groups. The NIHSS sub-item scores for consciousness [1.00 (0.00–4.00) vs. 0.00 (0.00–2.00), *p* < 0.001], motor [8.00 (6.00–8.00) vs. 6.00 (4.00–8.00), *p* < 0.001], and language [2.00 (0.00–3.00) vs. 1.00 (0.00–3.00), *p* = 0.004] were higher in the non-independence group compared to the independence group. Gaze sub-item scores also differed significantly between the two groups. The patients were also categorized into hemorrhage and no hemorrhage groups based on the occurrence of intracranial hemorrhage within 24 h of EVT. Motor scores were higher in the hemorrhage group [8.00 (6.00–8.00)] compared to the no hemorrhage group [6.00 (4.00–8.00)], *p* < 0.001. Gaze scores also differed significantly between the groups. Patients who died within 90 days had higher scores at baseline in the consciousness, gaze, facial palsy, motor, and language sub-items (Table 2).

**Table 2:** Sub-items of NIHSS of patients between different outcome groups.

Item	Functional independence	Non-functional independence	*p*	No intracranial hemorrhage	Intracranial hemorrhage	*p*	Mortality	Survival	*p*
Consciousness	0.00 (0.00–2.00)	1.00 (0.00–4.00)	<0.001	1.00 (0.00–4.00)	1.00 (0.00–4.00)	0.240	4.00 (0.75–5.00)	1.00 (0.00–3.00)	<0.001
Gaze	1.00 (0.00–2.00)	1.00 (0.00–2.00)	0.049	1.00 (0.00–2.00)	1.00 (0.00–2.00)	0.009	2.00 (1.00–2.00)	1.00 (0.00–2.00)	0.004
Visual fields	–	–	–	–	–	–	–	–	–
Facial palsy	1.00 (1.00–2.00)	2.00 (1.00–2.00)	0.380	1.00 (1.00–2.00)	1.00 (1.00–2.00)	0.995	2.00 (1.00–2.00)	1.00 (1.00–2.00)	0.001
Motor	6.00 (4.00–8.00)	8.00 (6.00–8.00)	<0.001	6.00 (4.00–8.00)	8.00 (6.00–8.00)	<0.001	8.00 (7.00–8.00)	7.00 (5.00–8.00)	<0.001
Ataxia	–	–	–	–	–	–	–	–	–
Sensory	–	–	–	–	–	–	–	–	–
Language	1.00 (0.00–3.00)	2.00 (0.00–3.00)	0.004	2.00 (0.00–3.00)	1.50 (0.00–3.00)	0.634	2.50 (0.00–3.00)	2.00 (0.00–3.00)	0.008
Dysarthria	0.00 (0.00–1.00)	0.00 (0.00–1.00)	0.299	0.00 (0.00–1.00)	0.00 (0.00–1.00)	0.141	0.00 (0.00–1.00)	0.00 (0.00–1.00)	0.650
Extinction/inattention	–	–	–	–	–	–	–	–	–

### Association between NIHSS sub-item scores and outcomes

**Figure 2:**
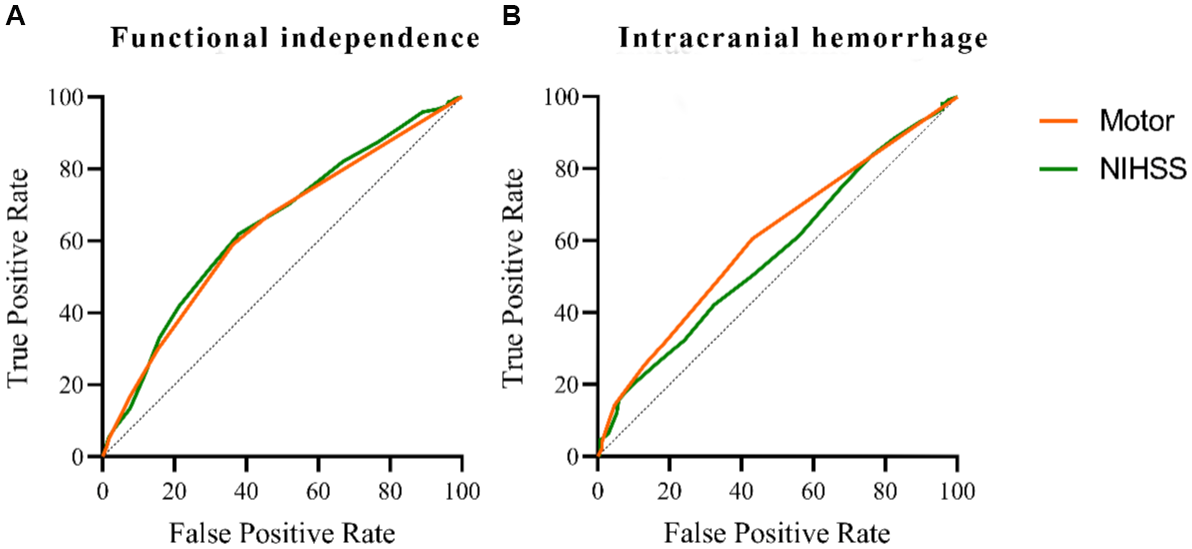
Comparison of predictive power among the NIHSS and motor score on functional independence at 3 months in patients with acute anterior circulation ischemic stroke receiving EVT. The area under the curve of the motor is 0.630, and the area under the curve of the NIHSS is 0.641 **(A)**. Comparison of predictive power among the NIHSS and motor score on intracranial hemorrhage within 24 h in patients with acute anterior circulation ischemic stroke receiving EVT. The area under the curve of the motor is 0.606, and the area under the curve of NIHSS is 0.568 **(B)**.

**Figure 3:**
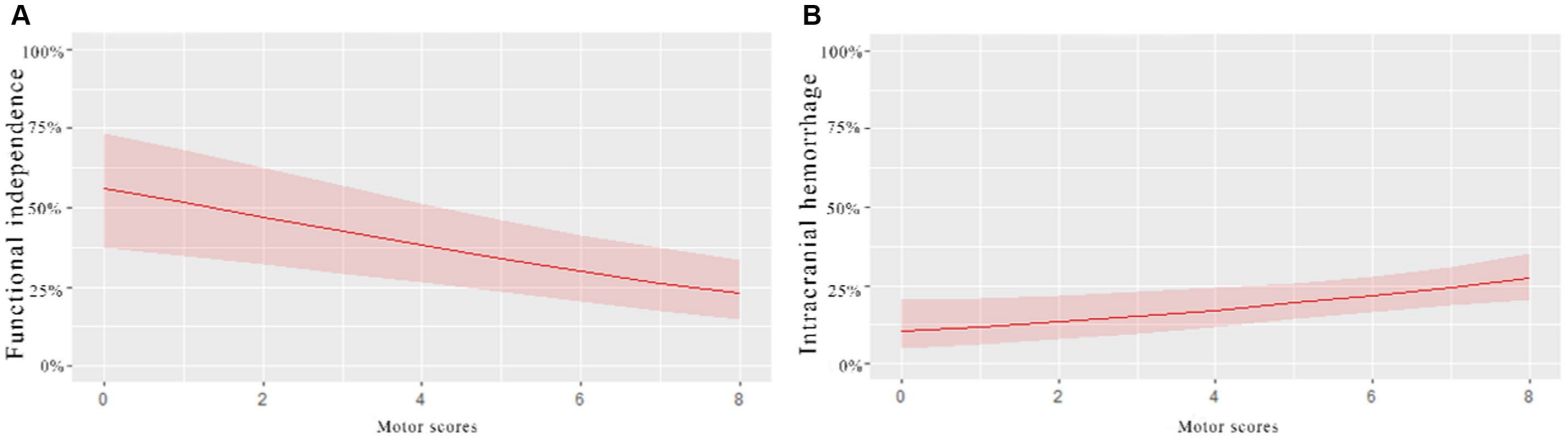
Association of motor scores with probabilities of different outcomes in patients with acute anterior circulation ischemic stroke receiving EVT. The **(A)**-curve shows that the higher the motor scores, the lower the probability of functional independence at 3 months. **(B)**-curve shows that the probability of intracranial hemorrhage within 24 h increased with the increase in the motor scores.

A univariate analysis found that the NIHSS sub-item scores for consciousness, gaze, motor, and language were associated with a 3-month prognosis after EVT. Gaze and motor sub-item scores were also associated with intracranial hemorrhage within 24 h of EVT. Figure 2 compares the predictive ability of motor sub-item scores vs. total NIHSS scores for the different outcomes. In a multivariate analysis adjusted for relevant factors, higher motor sub-item scores independently predicted lower odds of functional independence at 3 months (OR 0.833, 95% CI 0.758–0.915) and higher odds of hemorrhage within 24 h of EVT (OR 1.161, 95% CI 1.037–1.300) (Table 3). In patients who died within 3 months, consciousness (OR 1.351, 95% CI 1.122–1.614), gaze (OR 1.446, 95% CI 1.028–2.065), and facial palsy (OR 1.642, 95% CI 1.165–2.367) were associated with death, while motor (OR 1.137, 95% CI 0.949–1.390) was not. Figure 3 shows the association between motor sub-item scores and the predicted probabilities of different outcomes. Higher motor sub-item scores were associated with a decreased probability of a good functional outcome and an increased probability of intracranial hemorrhage.

**Table 3:** Association between sub-items of NIHSS and outcomes.

Item	Functional independence at 3 months				Intracranial hemorrhage at 24 h				Mortality within 3 months			
	Unadjusted OR (95%CI)	*p*	Adjusted OR^a^ (95%CI)	*p*	Unadjusted OR (95%CI)	*p*	Adjusted OR^b^ (95%CI)	*p*	Unadjusted OR (95%CI)	*p*	Adjusted OR^c^ (95%CI)	*p*
Consciousness	0.837 (0.768–0.912)	<0.001	0.877 (0.765–1.007)	0.062	–	–	–	–	1.335 (1.187–1.506)	<0.001	1.351 (1.122–1.641)	0.002
Gaze	0.830 (0.688–1.001)	0.052	0.985 (0.793–1.225)	0.893	1.312 (1.069–1.609)	0.009	1.101 (0.872–1.389)	0.420	1.567 (1.156–2.157)	0.005	1.446 (1.028–2.065)	0.038
Facial palsy	–	–	–	–	–	–	–	–	1.841 (1.309–2.651)	0.001	1.642 (1.165–2.367)	0.006
Motor	0.802 (0.738–0.871)	<0.001	0.833 (0.758–0.915)	<0.001	1.229 (1.112–1.358)	<0.001	1.161 (1.037–1.300)	0.009	1.301 (1.113–1.557)	0.002	1.137 (0.949–1.390)	0.183
Language	0.832 (0.733–0.944)	0.004	1.030 (0.843–1.259)	0.770	–	–	–	–	1.315 (1.075–1.622)	0.009	0.855 (0.613–1.180)	0.347

OR, odds ratio; CI, confidence interval.

a Adjust for age, sex, diabetes, time from arterial puncture to reperfusion or procedure completion, eTICI grade, baseline ASPECTS score, consciousness, gaze, motor, and language.

b Adjust for age, sex, diabetes, atrial fibrillation, time from arterial puncture to reperfusion or procedure completion, TOAST classification, baseline ASPECTS score, motor, and gaze.

c Adjust for age, sex, time from arterial puncture to reperfusion or procedure completion, eTICI grade, consciousness, gaze, facial palsy, motor, and language.

### Sensitivity analysis

In patients with a baseline NIHSS score of less than 13, between the low and high motor score groups (< 7 or ≥ 7 on motor scores), there was a significant difference in the proportion of functional independence [116 (78.4%) vs. 32 (21.6%); OR 0.539, 95% CI 0.297–0.976; *p* = 0.041] and intracranial hemorrhage [46 (63%) vs. 27 (37%); OR 2.206, 95% CI 1.162–4.186; *p* = 0.015].

## Discussion

This study examined the association between NIHSS sub-item scores and prognosis and hemorrhage outcomes in patients with anterior circulation ischemic stroke undergoing EVT. To our knowledge, this is the first study to evaluate correlations between individual NIHSS sub-item scores and intracranial hemorrhage after EVT. This is also the largest study to date assessing the association between NIHSS sub-item scores and prognosis following EVT. Our study demonstrated that among the many sub-items of NIHSS, the motor scores independently predicted prognosis at 3 months and intracranial hemorrhage within 24 h after EVT. Higher motor sub-item scores were associated with a poorer prognosis and increased hemorrhage risk. These findings can help emergency clinicians rapidly identify potential EVT candidates.

The NIHSS score is the most widely used tool for evaluating stroke severity (18, 19). Prior studies show that NIHSS scores correlate with collateral circulation and perfusion status (20–22). Higher admission NIHSS scores are associated with poorer outcomes following EVT in several studies (6, 23). However, the total NIHSS score may not precisely reflect cerebral vascular status and final infarct volume in acute ischemic stroke. For example, Mak et al. found larger infarct volumes in right vs. left hemisphere strokes despite equivalent total NIHSS scores (24). Hence, more representative scoring systems are needed to accurately evaluate these patients.

A prior study of 200 patients found that NIHSS scores were associated with the prognosis after EVT for anterior circulation stroke, with the consciousness sub-item playing a key role (25). Other studies have identified NIHSS components, including neglect, aphasia, facial palsy, and visual deficits, as predictors of poor outcomes or collateral status (20, 26, 27). In contrast to the above study, we found that motor scores were the strongest NIHSS predictor of 3-month prognosis (OR 0.833, 95% CI 0.758–0.915) in our larger cohort of 568 EVT patients.

The possible reason could be that motor scores comprise a major component of the total NIHSS. Higher motor scores typically indicate more severe and extensive infarcts in patients with large vessel occlusion stroke (28). In addition, the possible underlying mechanisms may involve differences in neuronal composition and neurovascular units between brain regions. The motor cortical areas contain large Betz cells that are more vulnerable to ischemia. Cortical regions also have a higher density of excitatory glutamatergic neurons and excitatory synapses that can exacerbate ischemic injury (29, 30). In addition, the motor cortex is supplied by terminal arteries and has less collateral flow compared to other cortical areas (31). These factors may contribute to the association between motor deficits and a poorer prognosis. Notably, motor scores can rapidly identify EVT candidates in the emergency department. However, total NIHSS and subtler sub-item deficits warrant comprehensive evaluation given their prognostic importance.

Hemorrhagic transformation is a common complication following EVT for acute ischemic stroke (32). Hemorrhage can lead to early neurological deterioration and poorer functional outcomes (33). Previous studies have found that factors associated with intracranial hemorrhage after EVT include higher admission NIHSS score, higher admission blood glucose, atrial fibrillation, oral anticoagulants, use of intravenous thrombolysis, longer time from onset to femoral artery puncture, and thrombolysis >3 times (32, 34, 35). Tian’s study found that receiving intravenous thrombolysis did not increase the risk of symptomatic intracranial hemorrhage (36). However, no study has specifically examined associations between individual NIHSS sub-item scores and hemorrhage following EVT, to our knowledge. In this study, 20 (3.5%) patients had symptomatic intracranial hemorrhage, while 150 (26.4%) had asymptomatic intracranial hemorrhage. Higher motor scores increased the odds of hemorrhage after EVT (OR 1.161, 95% CI 1.037–1.300), especially in patients with NIHSS scores less than 13 (OR 1.252, 95% CI 1.077–1.456). However, we found no significant association between baseline NIHSS and symptomatic intracranial hemorrhage (OR 1.059, 95% CI 0.009–1.129). Further study on the association between baseline NIHSS and NIHSS sub-items and symptomatic vs. asymptomatic hemorrhage is needed.

A mortality rate of 11.27% within 3 months was observed in this study. Compared to the mortality rate of 15.8% reported in the literature (6), this is slightly lower. The higher mortality may be attributable to factors such as higher baseline NIHSS scores, medical complications during the hospital, occlusion location, and unsuccessful recanalization (37, 38). Additionally, post-operative hemorrhagic complications could potentially contribute to the increased risk of mortality (37). Future studies should further investigate the linkage between post-procedural intracranial hemorrhage and mortality and prognosis. It also alludes to the potential value of further optimizing surgical techniques and reducing post-operative intracranial hemorrhage.

The study has several limitations. First, this is a retrospective study, and certain biases were inevitable. Second, we included all patients with acute anterior circulation ischemic stroke who received arterial thrombolysis and mechanical thrombectomy, without specific classification. Third, our findings are limited to anterior circulation strokes, and further study is needed to examine posterior circulation infarcts. Fourth, we only analyzed NIHSS sub-items, while combinations with other prognostic factors warrant further prospective investigation. Finally, specific details on intracranial hemorrhage subtypes were not recorded in this retrospective study. Tracking details such as petechial vs. parenchymal intracranial hemorrhage location in relation to vessel occlusion could better elucidate the mechanisms and clinical impact of hemorrhagic transformations after EVT; this can be further explored in the future.

## Conclusion

Higher limb motor sub-item scores on the NIHSS independently predicted a poorer prognosis at 3 months and an increased risk of intracranial hemorrhage within 24 h after EVT among patients with acute anterior circulation ischemic stroke. These findings can help emergency clinicians rapidly identify potential EVT candidates.

## Data availability statement

The raw data supporting the conclusions of this article will be made available by the authors, without undue reservation.

## Ethics statement

The studies involving humans were approved by the Ethics Committee of the First Hospital of Jilin University. The studies were conducted in accordance with the local legislation and institutional requirements. The ethics committee/institutional review board waived the requirement of written informed consent for participation from the participants or the participants' legal guardians/next of kin because the anonymous analysis of clinical data.

## Author contributions

SQ: Conceptualization, Formal analysis, Methodology, Writing – original draft. MS: Conceptualization, Writing – original draft. CL: Formal analysis, Writing – original draft. KS: Conceptualization, Formal analysis, Writing – original draft. JZ: Conceptualization, Methodology, Writing – original draft. FY: Formal analysis, Methodology, Writing – original draft. WZ: Conceptualization, Methodology, Writing – original draft. SW: Conceptualization, Data curation, Methodology, Writing – review & editing.
